# A quantitative trait locus analysis of personality in wild bighorn sheep

**DOI:** 10.1002/ece3.468

**Published:** 2013-01-18

**Authors:** J Poissant, D Réale, JGA Martin, M Festa-Bianchet, DW Coltman

**Affiliations:** 1 Department of Animal and Plant Sciences, University of SheffieldSheffield, S10 2TN, UK; 2 Département des Sciences Biologiques, Université du Québec à MontréalMontréal, Québec, H2X 1Y4, Canada; 3 Department of Ecology and Evolutionary Biology, University of California – Los AngelesLos Angeles, California, 90095-1606; 4 Département de Biologie, Université de SherbrookeSherbrooke, Québec, J1K 2R1, Canada; 5 Department of Biological Sciences, University of AlbertaEdmonton, Alberta, T6G 2E9, Canada

**Keywords:** Animal model, behavioral syndrome, boldness, docility, heritability, temperament

## Abstract

Personality, the presence of persistent behav105ioral differences among individuals over time or contexts, potentially has important ecological and evolutionary consequences. However, a lack of knowledge about its genetic architecture limits our ability to understand its origin, evolution, and maintenance. Here, we report on a genome-wide quantitative trait locus (QTL) analysis for two personality traits, docility and boldness, in free-living female bighorn sheep from Ram Mountain, Alberta, Canada. Our variance component linkage analysis based on 238 microsatellite loci genotyped in 310 pedigreed individuals identified suggestive docility and boldness QTL on sheep chromosome 2 and 6, respectively. A lack of QTL overlap indicated that genetic covariance between traits was not modulated by pleiotropic effects at a major locus and may instead result from linkage disequilibrium or pleiotropic effects at QTL of small effects. To our knowledge, this study represents the first attempt to dissect the genetic architecture of personality in a free-living wildlife population, an important step toward understanding the link between molecular genetic variation in personality and fitness and the evolutionary processes maintaining this variation.

## Introduction

Personality can be defined as the presence of persistent behavioral differences among individuals over time or contexts (Réale et al. 2007). Research in a number of organisms suggests that variability in personality may often have a genetic basis (van Oers et al. 2005) and contribute to variation in fitness (Dingemanse and Réale 2005; Smith and Blumstein 2008). Personality traits may therefore have important ecological and evolutionary consequences (Sih et al. 2004; Bell 2007; Réale et al. 2007; Wolf and Weissing 2012).

Knowledge about the genetic architecture of personality is a key to understand its origin, evolution, and maintenance (van Oers and Mueller 2010). Currently, most of our knowledge on the genetics of personality comes from studies in humans and a handful of laboratory model species, such as *Mus musculus* and *Drosophila melanogaster* (Kendler and Greenspan 2006; van Oers and Mueller 2010). Extending this research to nonmodel organisms, particularly wild species that are the focus of long-term individual-based field studies (see overview in Clutton-Brock and Sheldon 2010), would offer a unique opportunity to simultaneously study the link between molecular genetic variation in personality and fitness as well as micro-evolution in natural environments (Ellegren and Sheldon 2008; Kruuk et al. 2008; van Oers and Mueller 2010; Slate et al. 2010).

**Fig 1:**
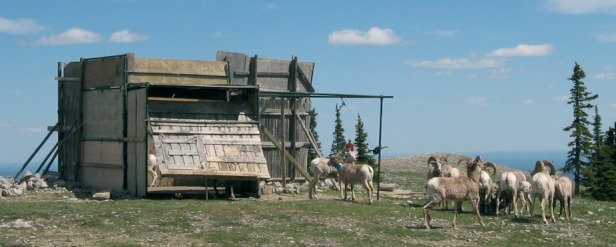
Corral trap baited with salt used to capture bighorn sheep at Ram Mountain, Alberta, Canada.

The bighorn sheep (*Ovis canadensis*) is a mountain ungulate endemic to western North America (Valdez and Krausman 1999). Personality has been studied in this species for over a decade (Réale et al. 2000, 2009; Réale and Festa-Bianchet 2003). Boldness (bold-shy continuum) and docility (docile-aggressive continuum) have been measured for most females residing at Ram Mountain, Alberta, Canada, using an index of trappability and the reaction of animals during captures in a corral trap baited with salt (Figure 1; see methods and Réale et al. 2000 for details). Pedigree-based quantitative genetic analyses determined that both traits had an additive genetic basis and were negatively genetically correlated (Réale et al. 2009). The availability of detailed life history information for most individuals also provided links between personality and fitness components. For example, bold and docile ewes were found to reach sexual maturity before shy and aggressive ones (Réale et al. 2000). Boldness in ewes was also positively correlated with weaning success (Réale et al. 2000) and survival in years of high cougar (*Puma concolor*) predation (Réale and Festa-Bianchet 2003). In males, docility and boldness were found to have a positive effect on longevity, a weak negative effect on early life reproductive success, and a strong positive effect on late life reproductive success (Réale et al. 2009). Personality therefore appears to play important roles in the ecology and evolution of this species.

In this study, we performed a genome-wide quantitative trait locus (QTL) scan for boldness and docility in Ram Mountain female bighorn sheep. In addition to test for the presence of QTL influencing each trait individually, we tested for the presence of QTL potentially modulating genetic covariance between traits. This represents an important step toward understanding the link between molecular genetic variation in personality and fitness and the evolutionary processes maintaining this variation.

## Methods

### Study population

The Ram Mountain bighorn sheep population is native to a small isolated mountain range located about 30 km east of the Canadian Rockies in Alberta, Canada (52°N, 115°W, elevation 1080–2170 m). Techniques used to capture, mark, measure, and monitor animals were described in detail by Jorgenson et al. (1993) and Réale et al. (2000, 2009). Briefly, animals were captured in a corral trap baited with salt (Fig. 1) from late May to September or early October each year. Sheep were caught by the horns, blindfolded and hog-tied in the trap before being dragged to a net outside the trap where various measurements (e.g., weight and horn size) and samples (e.g., biopsies for genetic analysis) were taken. Animals were marked in the year of birth, so their exact age was known. Marked sheep were subsequently monitored throughout their lifetime.

### Behavioral data

#### Docility

Docility was determined using the response of animals toward handlers from when they were caught by the horns to when they were hog-tied and wrapped in a net for weighing (Réale et al. 2000, 2009). Scores ranged from 0 to 7 for least to most docile animals, respectively. We only considered docility scores of adult females (2 years old and older) because males and younger females were rarely assessed. Analyses were based on docility measured from 1998 to 2011.

#### Boldness

Boldness was determined by counting the number of times an animal was handled in a field season (Réale et al. 2000, 2009). In brief, we assumed the number of captures to reflect an individual's acceptance of the risks involved in licking salt (i.e., being trapped and handled). Individuals frequently captured were therefore considered bold, whereas individuals rarely captured were considered shy. Boldness scores were often lower than the absolute number of captures because animals captured within 3 weeks of being handled were usually released without handling. We only measured boldness from the year in which an animal was first handled as an adult (at least 2 years old) to prevent comparing docility and boldness at different ontogenetic stages and because lambs and yearlings may unknowingly follow adults into the trap. We excluded the last record of animals removed from the study area (translocated or shot) before September 1^st^ and of animals that were not captured during their last year of life to limit the inclusion of low boldness scores resulting from removal or early season mortality. Male boldness scores were not considered because adult males only visited the trap early in the field season. This study is based on boldness scores calculated from 1992 to 2011. Earlier data were not included due to the smaller number of captures performed in those early years. Phenotypic variance was also artificially reduced from 1986 to 1991 when high population density made handling animals more than once a month impractical.

### Pedigree information

Pedigree reconstruction methods were described in detail in Poissant et al. (2012). In brief, maternity was inferred accurately in the field since the early 1970s using suckling behavior. Since 1988, collection of DNA samples allowed for genetically based maternity and paternity assignments. These later analyses were based on ∼30 microsatellite loci and the 95% confidence threshold in Cervus (Marshall et al. 1998). In addition, the software Colony (Wang 2004) was used to infer sibships resulting from sires without a DNA sample. The accuracy of parts of the pedigree was also recently assessed using >200 microsatellite loci used to construct a genetic linkage map (Poissant et al. 2009, 2010). The pedigree used in this study contains 822 maternal links resulting from 243 dams (mean number of offspring ±1 standard deviation [SD] = 3.38 ± 2.53) and 486 paternal links resulting from 72 sampled and 37 unsampled sires (mean number of offspring per sire = 4.46 ± 4.53).

Only parts of the pedigree were informative for QTL mapping because genotypes were only obtained for a subset of individuals. We therefore based our QTL mapping analyses on a restricted pedigree composed of 310 fully typed animals as well as a few individuals connecting fully typed animals that were either untyped or only typed at markers used for initial parentage analyses (n = 19 and 41, respectively). This pedigree was the same as the one used in Poissant et al. (2012) with one additional unsampled sire identified using Colony. The QTL mapping pedigree contained 210 females and 160 males connected by 301 maternal links (mean number of offspring per dam ±1 SD = 2.59 ± 1.49) and 269 paternal links (mean number of offspring per sire = 4.14 ± 3.33). The number of individuals with docility and boldness measurements ranged from 137 to 175 in the full pedigree, and from 77 to 94 in the QTL mapping pedigree (Table 1).

**Table 1:** Proportion of phenotypic variance after having accounted for fixed effects (*V*_p_) explained by additive genetic (*h*^2^), year, and permanent environmental effects for docility and boldness in adult bighorn sheep estimated using the full and the smaller QTL mapping pedigree

trait	dataset	ind.	obs.	mean (sd)	*V*_p_	*h*^2^	year	perm.env.
docility	full	94	1440	5.52 (1.62)	2.83 (0.28)	0.31 (0.16)*	0.04 (0.02)^***^	0.24 (0.14)*
	QTL	77	1311	5.60 (1.58)	2.79 (0.31)	0.24 (0.20)	0.04 (0.02)^***^	0.33 (0.18)*
boldness	full	175	867	3.95 (1.30)	1.68 (0.16)	0.21 (0.07)^***^	0.24 (0.07)^***^	0.03 (0.05)
	QTL	137	741	4.02 (1.29)	1.62 (0.17)	0.17 (0.07)^***^	0.27 (0.07)^***^	0.03 (0.05)

Number of individuals and observations included in each analysis as well as raw trait means (s.d. in parentheses) are also presented. Significance of ratios was assessed using likelihood ratio tests

* *P* < 0.05

^**^P < 0.01

^***^P < 0.001.

Standard errors generated by ASReml are presented in parentheses.

### Quantitative genetic analyses

Phenotypic variance in docility and boldness was partitioned into additive genetic and other components using the animal model (Lynch and Walsh 1998; Wilson et al. 2010) and restricted maximum likelihood implemented in ASReml 3.1 (Gilmour et al. 2009). Analyses were performed using untransformed as well as square-root transformed dependant variables because the distributions of phenotypes were skewed. Transformations did not affect conclusions qualitatively or quantitatively; therefore, we only present results from models based on untransformed variables. We used univariate models to estimate trait-specific parameters and bivariate models to estimate covariances and correlations. Univariate analyses of docility were based on individual measurements, whereas bivariate models to estimate genetic correlations between docility and boldness were based on yearly means of docility scores (yearly docility). We used yearly means in these models to allow us to estimate permanent environmental covariance. All analyses were performed using the full Ram Mountain pedigree as well as the more restricted QTL mapping pedigree for comparative purposes.

As in Réale et al. (2009), age was fitted as a continuous fixed effect in all models to account for ontogenetic changes in behavior. Docility increased linearly with age up to about 9 years, when it stabilized. We therefore pooled measurements of docility obtained from each sheep aged 9 and older. Boldness decreased linearly with age. Initial animal models included additive genetic, maternal environmental (mother identity), permanent environmental (identity), and year random effects. Maternal effects did not explain a large or significant amount of variation and were therefore excluded from final models. The proportion of phenotypic variance explained by mother identity was 0.06 ± 0.04 and 0.01 ± 0.12 for boldness and docility, respectively. The permanent environmental effect was fitted to account for inter-individual variation resulting from nongenetic causes as well as dominance and epistasis, and was retained in final models even when not significant as suggested by Wilson et al. (2010). Year was fitted to account for changes in field personnel, capture effort, and any other uncontrolled environmental or demographic effects on the rate of visitation at the trap. Phenotypic variance (*V*_p_) was therefore ultimately partitioned in four components after having taken fixed effects into account: additive genetic (*V*_a_), permanent environmental (*V*_pe_), year of capture (*V*_y_), and residual (*V*_r_). Heritability (*h*^2^) and other ratios were obtained by dividing individual variance components by *V*_p_, which was the sum of all variance components. Significance of (co)variance components and ratios was tested using likelihood ratio tests contrasting models, including and excluding individual random effects.

### QTL mapping

QTL mapping was performed by adding a QTL variance component (*V*_QTL_) to the animal model described above using pairwise estimates of identity-by-descent (IBD) for specific genomic locations (George et al. 2000; Slate 2005). In those models, *V*_p_ was therefore partitioned into five components (*V*_a_, *V*_QTL_*,V*_pe_, *V*_y_, and *V*_r_). IBD matrices were estimated every 1 cM (Haldane's mapping function) with the software Loki (Heath 1997) using information from the QTL mapping pedigree, genotypes from 238 microsatellite loci ordered along all 26 autosomes and the X chromosome, and map distances from the species integrated map described in Poissant et al. (2010). As in Beraldi et al. (2007a,b), IBD matrices for the X chromosome were estimated by treating the Y chromosome as a nonvariable X chromosome. After a burn-in period of 50 cycles, 1 million iterations were performed with statistics being stored every two iterations. Significance of QTL effects was determined using LOD scores calculated as


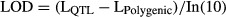


where L_QTL_ and L_polygenic_ were the log likelihood of models with and without a QTL component, respectively. As in Poissant et al. (2012), we adopted significance thresholds previously calculated for domestic sheep (*Ovis aries*) by Johnston et al. (2010) based on the formula from Lander and Kruglyak (1995). QTL were therefore considered suggestive and significant when LOD scores were greater than 1.88 and 3.31, respectively. Using thresholds inferred directly from the bighorn sheep map (1.83 and 3.27, respectively) would be anticonservative because the bighorn sheep map is missing markers at the end of chromosomes (Beraldi et al. 2006; Poissant et al. 2010). 95% CI for QTL positions were approximated using the one-LOD drop-off method of Lander and Botstein (1989) and a more conservative 1.5 LOD drop-off.

## Results

We partitioned phenotypic variance in docility and boldness into additive genetic, year, permanent environmental, and residual variance using the animal model (Table 1). Significant additive genetic variance was identified for both traits when considering the entire dataset, but only for boldness when considering the smaller QTL mapping dataset. Heritability estimates tended to be larger when estimated using the full dataset compared to the QTL mapping dataset (0.31 ± 0.16 vs. 0.24 ± 0.20 for docility and 0.21 ± 0.07 vs. 0.17 ± 0.07 for boldness). Year of sampling explained ∼5% of *V*_p_ for docility and ∼25% for boldness in both datasets. Finally, permanent environmental effects explained a large proportion of *V*_p_ for docility (∼25%), but were small and not significant for boldness.

Genetic correlations between docility and boldness were not significant for both the full and QTL mapping datasets (−0.36 ± 0.34 and −0.78 ± 0.44, respectively). Correlations for year and permanent environmental effects were 0.41 ± 0.28 and 0.20 ± 0.70 in the full dataset and 0.39 ± 0.29 and 0.58 ± 1.09 in the QTL mapping dataset, respectively.

**Fig 2:**
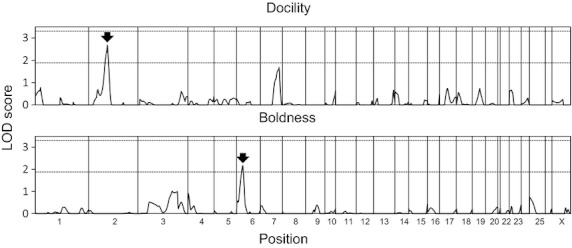
LOD scores along the 26 autosomes and the X chromosome for the presence of docility and boldness QTL in the Ram Mountain bighorn sheep population. Dashed horizontal lines depict the genome-wide suggestive (LOD > 1.88) and significant (LOD > 3.31) thresholds. Arrows highlight suggestive QTLs.

**Fig 3:**
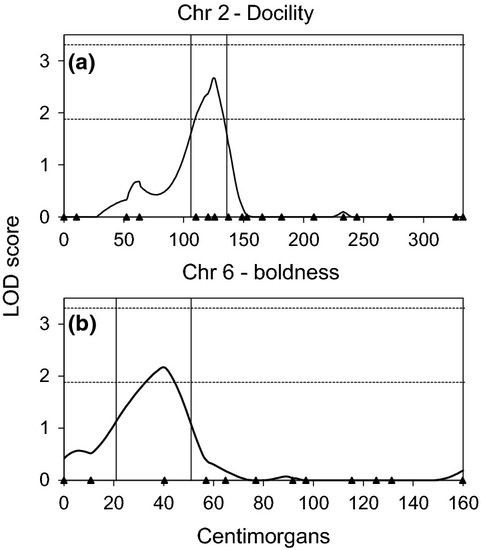
LOD scores along chromosomes on which suggestive QTL were detected in the Ram mountain bighorn sheep population. Dashed horizontal lines depict the conservative genome-wide suggestive (LOD > 1.88) and significant (LOD > 3.31) thresholds. The position of microsatellite markers along chromosomes are indicated by triangles along the x axis.

The genome scan identified two regions with LOD > 1.88, the threshold suggestive of significance (Fig. 2, Table 2). These putative QTL were located on chromosome 2 for docility and 6 for boldness. The 95% confidence interval estimated using the 1-LOD drop method spanned 30 cM for each QTL (Fig. 3). In both cases, the QTL appeared to explain all the additive genetic variance.

**Table 2:** Genomic position of putative QTL for personality traits in the Ram Mountain bighorn sheep population and their estimated parameters (*V*_QTL_, phenotypic variance explained by the QTL after having accounted for fixed effects; *q*^2^, proportion of phenotypic variance explained by the QTL after having accounted for fixed effects; *h*^2^, residual heritability after having fitted the QTL effect, as well as proportion of phenotypic variance explained by year and permanent environmental effects). Map distances are based on Haldane's mapping function and therefore not directly comparable to distances presented in Poissant et al. (2010) where Kosambi's mapping function was used. Standard errors generated by ASReml are presented in parentheses

Trait	LOD	Chr.	Pos. (cM)	Closest marker	1-LOD drop (cM)	1.5-LOD drop (cM)	*V*_QTL_	*q*^2^	*h*^*2*^	year	perm. env.
docility	2.67	2	125	BM4006	106-136	100-140	1.60 (0.46)	0.53 (0.09)	0.00 (-)	0.04 (0.02)	0.06 (0.06)
boldness	2.17	6	40	MCMA14	21-51	14-55	0.27 (0.09)	0.16 (0.05)	0.00 (-)	0.29 (0.07)	0.02 (0.03)

A visual comparison of trait-specific LOD scores on chromosome 2 and 6 did not reveal evidence for pleiotropic effects. In addition, LOD scores for docility and boldness never simultaneously exceeded nominal significance (*P* < 0.05, LOD > 0.5875) at the same position, but exceeded nominal significance at two different positions not far from each other at the distal end of chromosome 3.

## Discussion

We used a variance component approach to map QTL for boldness and docility and test for the presence of pleiotropic effects in a pedigreed population of bighorn sheep. To our knowledge, this represents the first attempt to dissect the genetic architecture of personality in a free-living wildlife population.

This study builds on previous quantitative genetic analyses in the same population by Réale et al. (2009). As expected, results differed slightly due to corrections and expansions of the phenotype-genotype dataset and modeling differences. For example, the additive genetic correlation between docility and boldness were similar in sign and magnitude between studies, but only significantly different from zero in Réale et al. (2009; −0.36 ± 0.34 in this study vs. −0.38 ± 0.15 in Réale et al. 2009). Another difference was a decrease in the heritability estimate for docility 0.31 ± 0.16 in this study versus 0.65 ± 0.06 in Réale et al. (2009). This decrease was attributable to the inclusion of a permanent environmental effects component in final models. Such effects were also apparent in previous analyses, but the absence of statistical significance made their inclusion in final models questionable (Réale et al. 2009). The larger sample size likely allowed us to detect significant permanent environmental effects. Permanent environmental effects could reflect a tendency of handlers to bias their evaluation toward expectations from past captures, permanent changes in animal behavior based on past experiences, or nonadditive genetic variation.

The QTL analysis did not reveal regions exceeding genome-wide significance (LOD > 3.31). The absence of significant QTL appear to be a characteristic of most QTL studies performed in outbred free-living wildlife populations to date (Slate et al. 2002; Beraldi et al. 2007a,b, Johnston et al. 2010; Tarka et al. 2010; Poissant et al. 2012). These results may reflect complex underlying genetic architectures, a lack of power, or both. Most traits in species, such as humans, flies, and mice appear to be influenced by a very large number of QTL of small effect (Kendler and Greenspan 2006; Flint and Mackay 2009; Munafo and Flint 2011; Yang et al. 2011). However, this pattern is not universal and traits, such as lateral plate variation in threespine sticklebacks (*Gasterosteus aculeatus*, Colosimo et al. 2005), horn size in Soay sheep (*Ovis aries*, Johnston et al. 2011), as well as size and shape variation in dogs (Boyko et al. 2010) appear to be influenced by genes of relatively large effect. In theory, the genetic architecture of quantitative traits should reflect selection patterns (Penke et al. 2007). For example, traits under balancing selection are expected to be influenced by a relatively small number of QTL of medium effect (Penke et al. 2007). This could be the case for personality in bighorn sheep, as there is evidence for fluctuating selection on female boldness through cougar predation (Réale and Festa-Bianchet 2003). Unfortunately, our current sample sizes do not allow us to estimate the proportion of variance explained by individual QTL, as evidenced by the fact that all QTL reaching suggestive significance were invariably attributed all the additive genetic variance (this study and Poissant et al. 2012). Such inflation of QTL effect sizes in small datasets is due to the selective reporting of significant results (Beavis 1998). It remains unclear what sample size would be needed to avoid the Beavis effect in complex pedigrees similar to the one used here. Simulations suggest that bias might persist even with data for more than 1000 individuals (J. Slate, pers. comm.).

Our linkage analysis identified two putative QTL regions surpassing the genome-wide suggestive significance threshold (LOD > 1.88). In a previous study on the genetic architecture of horn and body size in the same population, knowledge about QTL location for homologous traits in the closely related domestic sheep suggested that at least some nonsignificant QTL were true QTL (Poissant et al. 2012). Unfortunately, we are unaware of similar QTL studies for personality traits in domestic sheep. To our knowledge, cattle (*Bos taurus*) are the closest relative of bighorn sheep in which personality QTL have been mapped (Schmutz et al. 2001; Hiendleder et al. 2003; Gutierrez-Gil et al. 2008, Glenske et al. 2011) and a cross-species comparison did not yield evidence for overlapping QTL. Suggestive QTL regions in bighorn sheep nonetheless represent prime candidates for future studies. For example, a suggestive horn size QTL in domestic sheep was later found to be a major QTL (Johnston et al. 2010, 2011). Similarly, in zebra finch, suggestive QTL for wing length appeared to be nonrandomly associated with genes involved in feather development (Schielzeth et al. 2012).

Comparing results from the boldness and docility QTL scans did not reveal evidence for the presence of a major QTL having pleiotropic effect. This was consistent with the small (non significant) genetic covariance between traits and similar to findings from a study of temperament in cattle (Gutierrez-Gil et al. 2008). Genetic correlations among personality traits have been hypothesized to be controlled by a few master genes (van Oers and Mueller 2010). Our failure to detect such regions suggests that genetic covariance between docility and boldness in our study population may instead be influenced by linkage disequilibrium or genes of small effects. One such locus may be present in the distal end of chromosome 3 where LOD scores exceeded nominal significance for both traits (LOD > 0.5875). This region is homologous to cattle chromosome 5 where anxiety/docility QTL have been detected (Schmutz et al. 2001; Hiendleder et al. 2003) and contains the arginine vasopressin receptor 1A gene, which was linked to a number of social behavior and personality traits in mammals, including aggression, anxiety, and harm avoidance (Meyer-Lindenberg et al. 2009 and references therein). Obviously, this suggestion is highly speculative given the absence of genome-wide significance for any of the two traits in that region.

In this study, we used a large set of microsatellite markers to identify chromosomal regions potentially involved in personality variation in bighorn sheep. This is an important step toward linking molecular genetic variation in personality and fitness and studies of personality micro-evolution in this species (van Oers and Mueller 2010; Slate et al. 2010). Future study will include genotyping additional markers and archived DNA samples to provide better QTL mapping resolution, but also improving field protocols to reduce the amount of unexplained (residual) variation. It will also be useful to determine if genotype × environment effects are important, as accounting for such effects could improve QTL detection. Traits potentially genetically correlated with boldness and docility, such as fecal hormones or flight distance, could be used to validate QTL in other populations where animals cannot be captured. Finally, research on domestic sheep could help validate results as there is evidence for the conservation of genetic architectures between sheep species (Poissant et al. 2012).
